# Analysis of Suspected Food Poisoning Incidents in Riyadh, Saudi Arabia: An Epidemiological Perspective

**DOI:** 10.1155/ijm/4803121

**Published:** 2025-11-17

**Authors:** Abdullah A. Alajlan, Ayidh Almansour, Omar A. Alhumaidan, Mohammad Y. Alwetaid, Ali A. Al-Shehri, Suliman M. Alajel, Manal Almusa, Najla A. Albaridi

**Affiliations:** ^1^Saudi Food & Drug Authority (SFDA), Riyadh, Saudi Arabia; ^2^Department of Health Science, College of Health and Rehabilitation, Princess Nourah Bint Abdulrahman University, Riyadh, Saudi Arabia

**Keywords:** bacterial contamination, food samples, foodborne illness

## Abstract

Foodborne diseases (FBDs) pose significant challenges to public health and the food industry worldwide, including Saudi Arabia, where rapid urbanization, changes in dietary habits, and a growing food service sector have increased the risk of contamination. As a result, this study analyzed bacterial contamination in food samples from Riyadh, Saudi Arabia, and investigated food poisoning outbreaks in the city. A total of 7897 food samples and swabs collected between 2015 and 2018 were analyzed for coliforms, *Salmonella*, *Staphylococcus aureus*, and *Bacillus cereus*. Clinical data on food poisoning cases and outbreaks were also gathered to assess incidence rates and epidemiological trends. Bacterial identification followed ISO Standards and AOAC Official Methods. The results showed that 7.4% of the samples tested positive for the target pathogens: *Salmonella* (12.6%), *Bacillus cereus* (9.8%), and *Staphylococcus aureus* (3.4%). Females were found to have a slightly higher likelihood of food poisoning compared to males, with the highest incidence observed in the 20–49 age group. Common sources of foodborne illness included poultry (*n* = 93 cases), unclassified foods (*n* = 67), meat products (*n* = 45), rice (*n* = 38), vegetables (*n* = 36), and salads (*n* = 30). Food poisoning cases peaked in June, followed by April and August. This study highlights a significant gap in regional data on FBDs and emphasizes the need for improved surveillance and monitoring systems to reduce the incidence of foodborne illness in the region.

## 1. Introduction

### 1.1. Foodborne Disease (FBD)

FBD, commonly known as food poisoning, arises from the consumption of food containing various harmful agents. These agents encompass biological factors such as pathogenic microorganisms and their toxins (including bacteria, viruses, and parasites), chemical elements like heavy metals and other toxins (such as aflatoxin and cyanide), and physical elements such as glass particles [1, 2].

FBD causes common symptoms of food poisoning, including vomiting, diarrhea, stomach pains, and nausea. These symptoms typically appear within a short time of eating contaminated foods. Food can become contaminated at any stage in the food production process, from harvesting to consumption. Inadequate cooking, poor personal hygiene practices during food production and preparation, inappropriate storage temperatures, contaminated cooking equipment, and the use of food ingredients from contaminated sources are all possible mechanisms of food contamination [3]. Foodborne pathogens are often present in various foods, including raw or undercooked meat, poultry, seafood, eggs, unpasteurized dairy products, and fresh produce.

According to recent studies, the most prevalent foodborne pathogens are *Salmonella*, *Campylobacter*, and enterohaemorrhagic *Escherichia coli* [4]. Additionally, Beigh et al. [5] revealed that *Salmonella* contamination has become the second most common clinical manifestation of FBD in the country.

### 1.2. Incidence and Burden of FBD

FBDs are responsible for an estimated 600 million cases of illness and 420,000 deaths annually worldwide [6, 7]. According to the World Health Organization (2022), an estimated 40% of these deaths, approximately 125,000 annually, occur in children under 5 years of age [8]. In general, foodborne illness tends to be more severe in vulnerable groups, such as young children, the elderly, pregnant women, and people with weakened immune systems (*Centers for Disease Control and Prevention*, [9], and *Compendium of Microbiological Criteria for Food|Food Standards Australia New Zealand*, [10]). While several bacterial genera are responsible for the majority of FBD cases (*n* = 226,526,634 annually), viruses like norovirus (124,803,946 cases) and Hepatitis A (13,709,836 cases) also contribute significantly to the global burden, highlighting the considerable risk that viruses pose to public health [6, 11].

In the United States, the Centers for Disease Control (CDC) stated that roughly 48 million individuals had FBDs annually, resulting in 128,000 hospitalizations and 3000 deaths [12]. Furthermore, during 2020, the Saudi Ministry of Health (MOH) recorded 1270 cases of FBD (incidence rate: 2.34) and 134 instances of food poisoning [13]. However, the number of reported cases was 199 of FBD and 22 of food poisoning in the Riyadh region alone [13].

Although outbreaks of FBD are preventable, they affect the reputation of food companies and countries and result in huge economic losses, in particular through loss of productivity [14]. In low- and middle-income countries, hazardous food has been judged as responsible for annual economic losses of around US$110 billion from reduced productivity and increased medical bills [9]. Furthermore, there is evidence to suggest that the number of incidents of FBD has increased throughout the world [15]. Overall, therefore, regulatory authorities, governments, and food companies need to give more attention to ensuring that food does not become contaminated with agents that may cause outbreaks of FBD.

### 1.3. Strategies to Reduce the Incidence of FBDs: The Role of Surveillance and Prevention

On a global level, efforts to reduce food contamination and outbreaks of FBD flow from the Foodborne Disease Burden Epidemiology Reference Group (FERG), which was established by the World Health Organization (WHO) to estimate the global and regional burden of FBD [16]. On a national scale, initiatives have traditionally centered on providing comprehensive education and training to individuals along the food supply chain and on establishing systems to ensure that food manufacturers, regulatory authorities, and governments work together to improve the implementation of food laws and policies [3, 10]. Moreover, academic work can contribute to these efforts by identifying potential gaps and needs in the food surveillance system to ensure the development of national risk–based food safety measurement and efficient management of resources [17].

Another potential contribution of academic studies into patterns of FBD lies in the in-depth analysis of data on FBD outbreaks to estimate the gap between cases reported through surveillance systems and unreported cases. This work can help improve surveillance in the future [18], which can, in turn, help in the identification of public health priorities, estimation of the burden of FBD, estimation of the cost of FBD control measures, and investigation of disease prevention and FBD control programs (MOH, Saudi Arabia, n.d.; [19]). Moreover, surveillance systems are important for the efficient detection and response to FBD and for identifying emerging issues related to food safety and research needs. Information from the surveillance system acts as a reservoir to carry out risk assessment, for the development of risk management options and for the implementation of risk communication systems. Furthermore, these systems are important for identifying future FBD outbreaks [20].

### 1.4. Study Objective

Based on the background information provided above, the current study is aimed at analyzing and compiling data on FBD outbreaks in the Riyadh region from October 2015 to December 2018. This will help determine the trend, the primary pathogens involved, and the public health burden of FBDs during that time frame. It also attempts to assess the efficacy of the surveillance and prevention measures being implemented, pointing out potential flaws in the current framework for regional food safety management.

## 2. Materials and Methods

### 2.1. Study Design and Sampling Collection

In Saudi Arabia, four governmental committees are responsible for the investigation of FBD outbreaks, namely, the Ministry of Health (MOH), the Saudi Food and Drug Authority (SFDA), the Ministry of Municipal and Rural Affairs (MOMRA), and the Ministry of the Interior (MOI) [21].

The MOH is responsible for the examination of patients, as well as the determination of the history of illness and symptoms. The SFDA plays a significant role in cases involving suspected food poisoning by collecting samples from establishments under suspicion, conducting sample analysis, and pursuing legal action against wrongdoers. Meanwhile, MOMRA focuses on collecting samples from restaurant environments and verifying the licenses of both restaurant employees and the establishments themselves [22, 23]. The MOI is responsible for ensuring security and conducting investigations during FBD outbreaks. This multiplicity of actors, however, leaves room for gaps when addressing FBD outbreaks in Saudi Arabia.

The purpose of this study is to ascertain the true incidence rate by performing a thorough descriptive laboratory examination of foodborne occurrences that occurred in the Riyadh region between October 2015 and December 2018. Acquiring samples of questionable food is one of the SFDA's directives during the inquiry. As a result, SFDA inspectors often gather two kinds of samples: food samples and swabs of equipment used in restaurant preparation, such as trays, knives, and spoons. A laboratory information management system (LIMS) receives the findings of these tests, which are conducted in accordance with International Organization for Standardization (ISO) standard protocols. SFDA collected information for this study from the database from October 2015 to December 2018.

### 2.2. Descriptive Laboratory Analysis of FBD Incidences

This section focused on the most prevalent of foodborne pathogens, namely, *Salmonella* spp., *Staphylococcus aureus*, *Bacillus cereus*, and coliforms. Sample collection was conducted between October 2015 and December 2018 in Riyadh, Saudi Arabia. A total of 7897 food samples and swabs were collected randomly from different sources and analyzed.

Following the guidelines outlined in the Association of Official Analytical Chemists (AOAC) Official Methods and ISO Standards, bacteria were isolated from food samples and swabs. To isolate *Salmonella* species, ISO 6579-1, “Microbiology of the food chain – Horizontal method for the detection, enumeration, and serotyping of *Salmonella* – Part 1: Detection of *Salmonella* spp.,” was implemented. “Microbiology of food and animal feeding stuff – Horizontal method for the enumeration of presumptive *B. cereus* – Colony-count technique at 30°C” (ISO 7932) was followed in the isolation process of *B. cereus*. The instructions in ISO 6888-1, “Microbiology of food and animal feeding stuff – Horizontal method for the enumeration of coagulase-positive Staphylococci (*S. aureus* and other species),” were followed. Lastly, 3 M Petrifilm Coliform Count Plates are employed for the identification and enumeration of coliform bacteria.

All isolated bacteria were identified using matrix-assisted laser desorption/ionization (MALDI-TOF MS, Bruker), according to the manufacturer's instructions. Data analysis included characterization of samples by organism and the food matrix tested. Each food matrix is associated with a higher incidence of certain contaminating organisms. After this, we examined the prevalence of the four organisms of interest in various food matrices.

## 3. Results

### 3.1. Descriptive Laboratory Analysis

The rate of food poisoning incidents in Riyadh during the study period was 20 incidents every year. In addition, the total ratio of food poisoning incidents in Riyadh by population was 7.30 incidents for every 1 million of Riyadh's population over this period. Further, there was a nearly equal distribution of cases between females (50.3%) (*n* = 546) and males (49.7%) (*n* = 540), indicating no significant gender-based difference in the incidence of reported cases.

**Figure 1:**
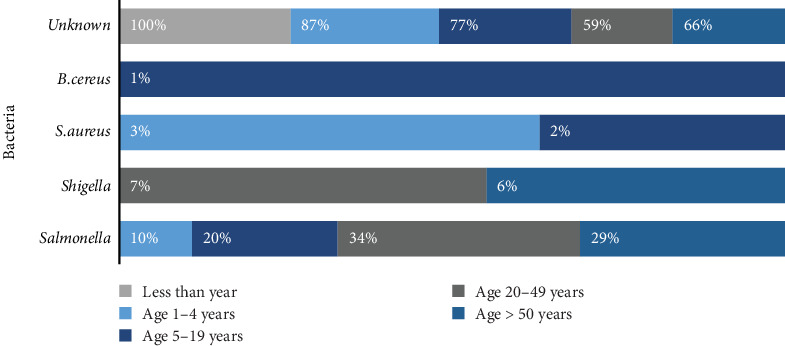
Age, etiology, and number of foodborne illness outbreaks recorded in Riyadh, 2015–2018.

Besides, there was a higher number of foodborne illness symptoms in the 20–49 age group, followed by the 1–4, 15–20, and then the more-than-50 age groups (Figure 1). The lowest number of incidents was in the less-than-1-year age group. Moreover, the investigation listed the following foods in order of frequency as the main vehicles of food poisoning cases: chicken (*n* = 93), meat items (*n* = 45), rice (*n* = 38), vegetables (*n* = 36), and salads (*n* = 30). In 67 cases, the food could not be identified.

**Figure 2:**
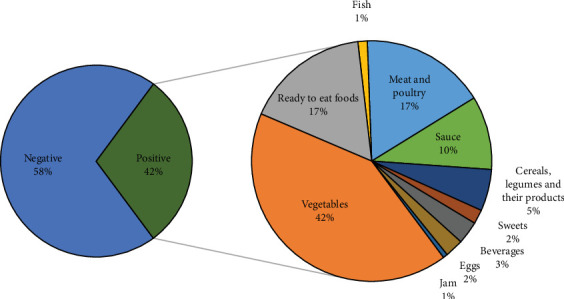
A pie-of-pie chart illustrating the distribution of coliform contamination across different food categories.

A total of 1707 food samples and swabs were tested out of 7897 collected across Riyadh between October 2015 and December 2018, representing 22% of the total samples. Of these, 7.4% were positive for the targeted pathogens. The food matrix of each positive sample was classified into several food categories according to the Standardization Organization for GCC (2015). Four bacterial species were isolated from food samples. According to the results, 161 out of 383 (42%) of the food samples and 9 out of 79 isolates from swabs (11.4%) were positive for coliforms, as shown in Figure 2. Notably, vegetables (cooked or noncooked) had the highest level of contamination by coliforms (42%), followed by meat and poultry (17%), ready-to-eat food (17%), sauces (10%), cereals (5%), beverages (3%), sweets (2%), and eggs (1%).

**Figure 3:**
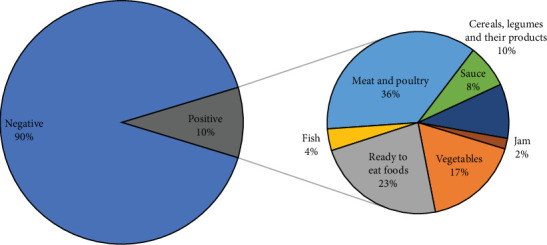
A pie-of-pie chart illustrating the distribution of *Salmonella* spp. contamination across different food categories.

Moreover, 52 out of 507 (10.3%) of food samples and 4 out of 176 (2.3%) of swabs were positive for *Salmonella* spp. (Figure 3). Furthermore, 36% of isolated *Salmonella* spp. were obtained from poultry and meat and 23% from ready-to-eat food, followed by vegetables (17%), cereals (10%), sauce (8%), fish (4%), and jam (2%) (Figure 2). On the other hand, the recorded number of food poisoning cases by *Salmonella* spp. was 280, of which 143 (51.1%) were males and 137 (48.9%) were females. In total, there were 19 *Salmonella*-associated outbreaks with 115 cases of hospitalization caused by *Salmonella* spp. Considering classification by age group, 169 cases were between 20 and 49 years old, followed by 5–19 years old, 10 cases were ≥50 years old, and 9 were between 1 and 4 years old (Figure 1). In January, August, and October, there were three *Salmonella*-related outbreaks each, whereas the remaining months experienced fewer than three outbreaks.

**Figure 4:**
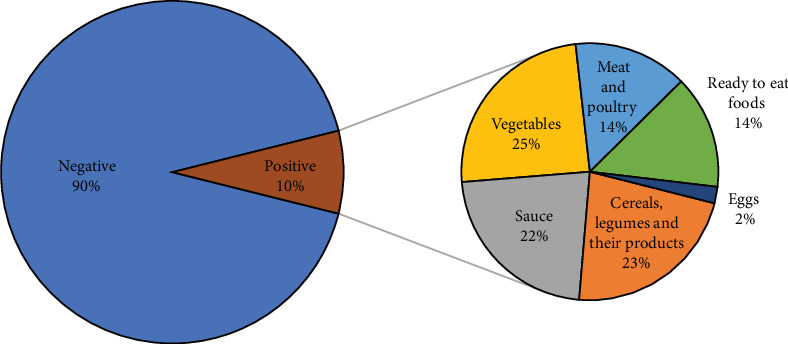
A pie-of-pie chart illustrating the distribution of *B. cereus* contamination across different food categories.

In contrast, *B. cereus* was isolated from 49 out of 500 samples (9.8%) (Figure 4). Our findings indicated that these isolates were found most in vegetables (25%), cereals (23%), sauces (22%), meat and poultry (14%), ready-to-eat food (14%), and eggs (2%). Additionally, four cases of food poisoning caused by *B. cereus* (two females and two males) were reported, with a single outbreak in March 2018 in the age group of 20–49 years.

**Figure 5:**
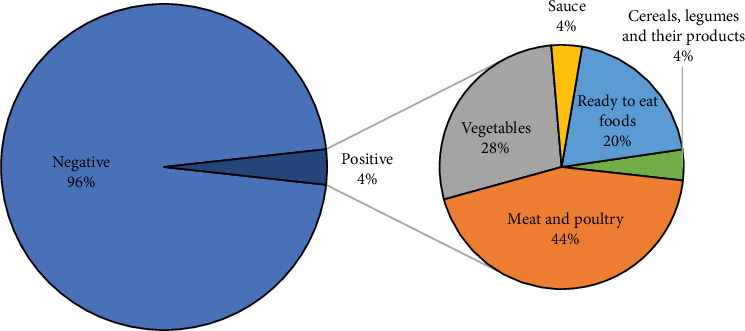
A pie-of-pie chart illustrating the distribution of *S. aureus* contamination across different food categories.

Additionally, out of the 700 isolates examined, 24 samples (3.4%) tested positive for *S. aureus* (Figure 5). S. aureus was present in 44% of meat and poultry, 28% of vegetables, 20% of ready-to-eat food, 4% of cereals, and 4% of sauce. Furthermore, *S. aureus* was implicated in 13 food poisoning cases (five males and eight females) from three outbreaks in August and April of 2018. Nine of those cases affected patients between 5 and 19 years old, three cases affected patients between 1 and 4 years old, and one case affected a patient between 20 and 49 years old.

**Figure 6:**
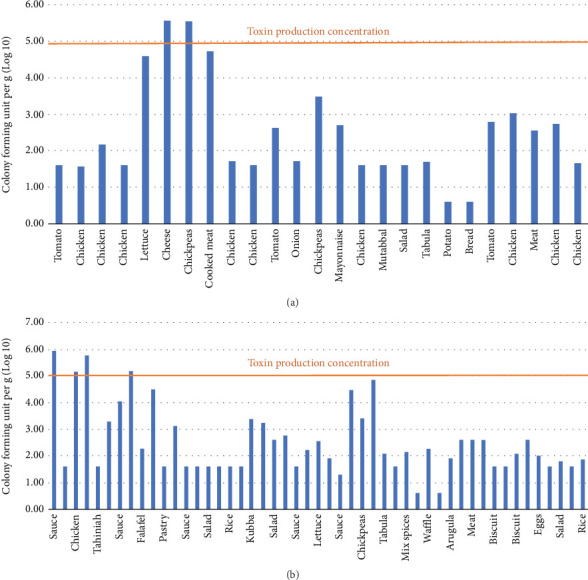
A bar graph demonstrating bacterial growth and toxin production concentrations: (a) *S. aureus* and (b) B. *cereus*.

Given that both *S. aureus* and *B. cereus* can produce toxins, it was critical to calculate the expected bacterial growth in order to assess whether the bacteria had attained the threshold required for the creation of toxins. Two *S. aureus* samples achieved the amount of toxin production, and two samples nearly achieved it (Figure 6a). Additionally, three *B. cereus* samples nearly approached the level of toxin production, whereas four samples reached it (Figure 6b).

**Figure 7:**
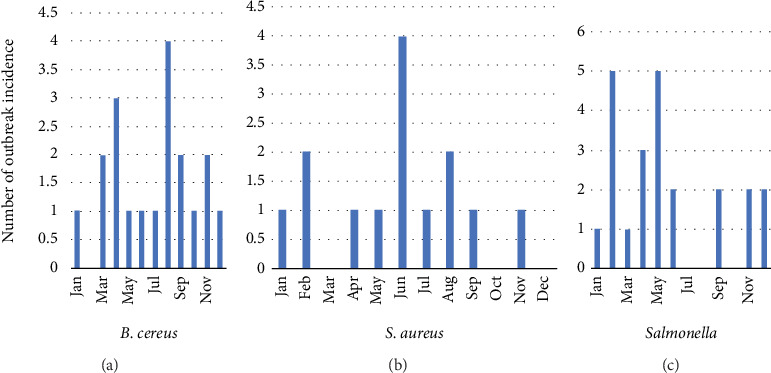
A bar chart showing the monthly record of confirmed food poisoning incidents in Riyadh from October 2015 to December 2018. (a) *B. cereus*, (b) *S. aureus*, and (c) *Salmonella* spp.

In the food poisoning cases, different swabs were taken from restaurants, such as knives and spoons, tables, and refrigerators. Such collected swabs were studied for the presence of coliforms, *Salmonella*, *S. aureus*, and *B. cereus*. Chronology was noted on the poisoning cases relating to *Salmonella* spp., *S. aureus*, and *B. cereus* (Figure 7). All the microbe targets were identified and enumerated according to their respective prevalence in each month. Lastly, the MALDI-TOF method was used to confirm positive isolates of *Salmonella* spp., *S. aureus*, and *B. cereus*.

### 3.2. Estimated Reported Food Illness Prediction Result

The laboratory analyses conducted between 2015 and 2018 in Riyadh revealed important insights into FBD outbreaks. The results, summarized in Table 1, include the number of reported outbreaks, cases, and deaths by etiology. To validate the model's accuracy, the scaled annual rate was used as a control value. Preliminary findings indicate that the annual reported rates of foodborne illnesses are as follows: 70 cases of *Salmonella* spp., 37 cases of *Shigella* spp., approximately three cases of *S. aureus*, and one case of *B. cereus*. All approximations and scaling were carried out as detailed in the previous section (Materials and Methods).

## 4. Discussion

This investigation into outbreaks of FBD in Saudi Arabia from 2015 to 2018 provides critical insight into the strengths and weaknesses of the current food safety system. While much effort has been directed toward surveillance and control regarding FBDs, this study indicated an area where the mechanisms of current surveillance and response may potentially be insufficient. These may influence the timeliness of outbreak identification and, through that, the effectiveness of the overall management of outbreaks for optimal public health outcomes.

Based on our results, only 7.4% of the 1707 analyzed samples carried the three pathogens of interest. To our knowledge, this prevalence is far less than what would be expected, based on the previous international literature. For instance, according to a prior US study, 51% of 5760 outbreaks between 2009 and 2015 had a single verified causal bacterium [7]. Meanwhile, the Shandong Province CDC, China, received reports of 1043 FBD outbreaks from 2011 to 2016 [8]. According to the same authors, 71.3% of those outbreaks were linked to an implicated food or contaminated ingredient [8]. The fact that our study observed a much lower percentage of samples containing identified causative organisms may be attributed to various factors. These factors include challenges in selecting the appropriate test for the suspected causative organism based on the associated symptoms of food poisoning cases or potential issues within the sampling procedures, such as delays between the notification of a suspected outbreak and the collection of samples.

Even with the lower rate of detection, the incidence of foodborne illness per million people in Riyadh is high, compared with other internationally reported rates. From the data in this study, a rate of 7.30 incidents per million population in Riyadh over the 3 years was calculated. By comparison, a rate of 2.6 outbreaks per million people was reported in 50 of the states within the United States [7]. The median number of incidents per year in Riyadh during the study period was 20. On the other hand, 69 outbreaks were annually reported in the Shandong Province CDC, China [8].

This study also sheds light on potential reasons for the comparatively high rate of outbreaks in Riyadh. The high rates of coliform bacteria in the vegetable samples could point to a lack of quality in the vegetables used or poor cleaning and handling before use.

The foods most contaminated by pathogenic bacteria were meat and poultry, which were usually contaminated with *Salmonella* and *S. aureus*. Based on the growth levels of *S. aureus* and *B. cereus* in the presented data, a small percentage of the samples reached the stage of toxin production. According to Food Standards Australia New Zealand (2018), *S. aureus* starts to produce toxin at the growth level of 10^5^ − 10^8^ cfu/g and *B. cereus* at a growth greater than 10^5^ cfu/g [24].

## 5. Conclusions

This research shows high incidents of cross-contamination in restaurants in Riyadh City because of a lack of stringent adherence to proper methods in the use of food utensils and handling to attain proper food safety practices during the period of investigation. Results show the desperate need for extensive training to make the personnel of different restaurants aware of how they could prepare food safely in appropriate personal hygiene to lessen contamination risks and maintain health within the public. Although there was no extreme seasonal variation in the notification of food poisoning cases, this consistent trend throughout the months manifests the continuity of those problems. It is clear that addressing these gaps in food safety practices will definitely be one way of minimizing foodborne illnesses and assuring the safety of customers consuming food from restaurants.

## Figures and Tables

**Table 1:** Summary of the descriptive results of the laboratory analyses, including the number of reported foodborne disease outbreaks, cases, and deaths by etiology in Riyadh between 2015 and 2018.

**Variables**	**No. of outbreaks**	**No. of cases**	**No. of hospitalization**	**No. of deaths**	**Scaled annual rate**		
					**Case per year** ^**a**^	**Outbreak per year**	**Case per one outbreak**
Pathogens							
*Salmonella* spp.	19	280	115	0	70	5	15
*Shigella* spp.	1	37	1	0	37	1 ^b^	37
*Staphylococcus aureus*	3	13	9	0	3.25^c^	1	4
*Bacillus cereus*	1	4	0	0	1	1	4

^a^All cases was divided by 4 years.

^b^The annual rate was rounded to be one outbreak per year, because the current rate cannot divided by 4.

^c^The case was rounded to 3 from 3.25.

## Data Availability

The data that support the findings of this study are available in the supplementary material of this article.

## References

[1] Al-Goblan A. S., Jahan S. (2010). Surveillance for Foodborne Illness Outbreaks in Qassim, Saudi Arabia, 2006. *Foodborne Pathogens and Disease*.

[2] Aljoudi A., Al-Mazam A., Choudhry A. (2010). Outbreak of Food Borne Salmonella Among Guests of a Wedding Ceremony: The Role of Cultural Factors. *Journal of Family and Community Medicine*.

[3] Al-Mohaithef M., Abidi S. T., Javed N. B., Alruwaili M., Abdelwahed A. Y. (2021). Knowledge of Safe Food Temperature Among Restaurant Supervisors in Dammam, Saudi Arabia. *Journal of Food Quality*.

[4] Al-Mutairi S., Connerton I., Dingwall R. (2014). Food Safety Organisations in Saudi Arabia–Organisational, Historical and Future Analysis. *Food Control*.

[5] Beigh S., Mahzari A., Alharbi R. A. (2023). A Retrospective Study of Epidemiological Correlations of Food, Drug and Chemical Poisoning in Al-Baha, Western Saudi Arabia. *Western Saudi Arabia. Healthcare*.

[6] Boxman I. L., Tilburg J. J., Te Loeke N. A., Vennema H., De Boer E., Koopmans M. (2007). An Efficient and Rapid Method for Recovery of Norovirus From Food Associated With Outbreaks of Gastroenteritis. *Journal of Food Protection*.

[7] Foodborne Disease Burden Epidemiology Reference Group (FERG) (2015). WHO Estimates of the Global Burden of Foodborne Diseases: Foodborne Diseases Burden Epidemiology Reference Group 2007-2015. https://www.who.int/publications/i/item/9789241565165.

[8] World Health Organization: WHO (2022). World Health Statistics 2022. https://www.who.int/news/item/20-05-2022-world-health-statistics-2022.

[9] Centers for Disease Control and Prevention. Estimates: Burden of foodborne illness in the United States. U.S. Department of Health & Human Services. https://www.cdc.gov/food-safety/php/data-research/foodborne-illness-burden/index.html.

[10] https://www.foodstandards.gov.au/publications/Compendium-of-Microbiological-Criteria-for-Food.

[11] Dewey-Mattia D., Manikonda K., Hall A. J., Wise M. E., Crowe S. J. (2018). Surveillance for Foodborne Disease Outbreaks — United States, 2009–2015. *MMWR Surveillance Summaries*.

[12] Scallan E., Hoekstra R. M., Angulo F. J. (2011). Foodborne Illness Acquired in the United States-Major Pathogens. *Emerging Infectious Diseases*.

[13] Sharaheeli J., Alibrahim H., Abd-Ellatif E. E. (2023). Evaluation of surveillance and response systems of foodborne diseases and outbreaks at regional level in Riyadh City-Saudi Arabia. *Journal of Community Medicine and Public Health Reports*.

[14] Gallo M., Ferrara L., Calogero A., Montesano D., Naviglio D. (2020). Relationships Between Food and Diseases: What to Know to Ensure Food Safety. *Food Research International*.

[15] Hachemi A., Zenia S., Denia M. F., Guessoum M., Hachemi M. M., Ait-Oudhia K. (2019). Epidemiological Study of Sausage in Algeria: Prevalence, Quality Assessment, and Antibiotic Resistance of Staphylococcus aureus Isolates and the Risk Factors Associated With Consumer Habits Affecting Foodborne Poisoning. *Veterinary World*.

[16] Hardnett F. P., Hoekstra R. M., Kennedy M., Charles L., Angulo F. J., the Emerging Infections Program FoodNet Working Group (2004). Epidemiologic Issues in Study Design and Data Analysis Related to FoodNet Activities. *Clinical Infectious Diseases*.

[17] Havelaar A. H., Kirk M. D., Torgerson P. R. (2015). World Health Organization Global Estimates and Regional Comparisons of the Burden of Foodborne Disease in 2010. *PLoS Medicine*.

[18] Koopmans M., Duizer E. (2004). Foodborne Viruses: An Emerging Problem. *International Journal of Food Microbiology*.

[19] Naeem S., Xie Y., Naeem S., Mubarik S., Yuan Z., Shi K. (2022). A Study Design to Determine Parents’ Knowledge, Attitude and Preventive Practice and Associated Factors to Combat Food Poisoning: A Cross-Sectional Survey From Lahore, Pakistan. *Journal of Food Security*.

[20] Pires S. M., Desta B. N., Mughini-Gras L. (2021). Burden of Foodborne Diseases: Think Global, Act Local. *Current Opinion in Food Science*.

[21] Sarma P. K., Alam M. J., Begum I. A. (2022). Red Meat Handlers’ Food Safety Knowledge, Attitudes, and Practices in the Dhaka Megacity of Bangladesh. *International Journal of Food Properties*.

[22] Todd E. C. (2017). Foodborne Disease and Food Control in the Gulf States. *Food Control*.

[23] Wu G., Yuan Q., Wang L. (2018). Epidemiology of Foodborne Disease Outbreaks From 2011 to 2016 in Shandong Province, China. *Medicine*.

[24] (2024). *Food Standards Australia New Zealand*. https://www.foodstandards.gov.au/.

